# BH3 profiling and a toolkit of BH3-mimetic drugs predict anti-apoptotic dependence of cancer cells

**DOI:** 10.1038/bjc.2016.49

**Published:** 2016-03-08

**Authors:** Michael Butterworth, Andrew Pettitt, Shankar Varadarajan, Gerald M Cohen

**Affiliations:** 1 Departments of Molecular and Clinical Cancer Medicine, University of Liverpool, Liverpool L69 3GE, UK; 2 Department of Pharmacology, University of Liverpool, Liverpool L69 3GE, UK

**Keywords:** BH3 profiling, A-1331852, A-1210477, ABT-199, MCL-1, BCL-XL

## Abstract

**Background:**

Anti-apoptotic BCL-2 family members antagonise apoptosis by sequestering their pro-apoptotic counterparts. The balance between the different BCL-2 family members forms the basis of BH3 profiling, a peptide-based technique used to predict chemosensitivity of cancer cells. Recent identification of cell-permeable, selective inhibitors of BCL-2, BCL-X_L_ and MCL-1, further facilitates the determination of the BCL-2 family dependency of cancer cells.

**Methods:**

We use BH3 profiling in combination with cell death analyses using a chemical inhibitor toolkit to assess chemosensitivity of cancer cells.

**Results:**

Both BH3 profiling and the inhibitor toolkit effectively predict chemosensitivity of cells addicted to a single anti-apoptotic protein but a combination of both techniques is more instructive when cell survival depends on more than one anti-apoptotic protein.

**Conclusions:**

The inhibitor toolkit provides a rapid, inexpensive and simple means to assess the chemosensitivity of tumour cells and in conjunction with BH3 profiling offers much potential in personalising cancer therapy.

A cardinal feature of cancer cells is their resistance to apoptosis. Cancer chemotherapeutic drugs induce apoptosis primarily by perturbation of mitochondrial integrity, which is regulated by the BCL-2 family of proteins. Anti-apoptotic BCL-2 family proteins, principally BCL-2, BCL-X_L_ and MCL-1, maintain survival of cancer cells by sequestering their pro-apoptotic counterparts. Although the literature abounds with many purported BCL-2 family inhibitors, few are specific (Vogler *et al*, 2009b; Varadarajan *et al*, 2013). Two specific inhibitors, navitoclax (ABT-263), which inhibits BCL-2, BCL-X_L_ and BCL-w, and venetoclax (ABT-199), which inhibits BCL-2, have recently entered clinical trials (Tse *et al*, 2008; Souers *et al*, 2013) and show promise in haematological malignancies, such as chronic lymphocytic leukaemia (CLL) (Roberts *et al*, 2015). Recently, A-1331852 and A-1210477 have been identified as specific inhibitors of BCL-X_L_ and MCL-1, respectively (Leverson *et al*, 2015a, 2015b). Importantly ABT-199, A-1331852 and A-1210477, are cell-permeable, thus permitting direct interrogation of cancer cells to ascertain the key proteins responsible for their survival.

BH3 profiling is a peptide-based technique used to predict the chemosensitivity of cancer cells and measures the ability of different BH3 peptides to induce mitochondrial depolarisation, which acts as a surrogate marker for the cellular response to chemotherapeutic agents (Ni Chonghaile *et al*, 2011). Recently Letai and co-workers established a related, dynamic BH3 profiling (DBP) to measure early changes in pro-apoptotic signalling following exposure to chemotherapeutic agents (Montero *et al*, 2015). In this communication, we compare BH3 profiling with a chemical toolkit comprising specific BCL-2 family inhibitors and assess additional benefits of employing them together to address chemoresistance and BCL-2 family dependence of various cancer cells.

## Materials and Methods

### Cell culture

Peripheral blood samples from CLL patients were obtained with patient consent and local ethics committee approval and cultured as described (Vogler *et al*, 2009b). MOLT-4 and H1299, an AML and non-small cell lung carcinoma cell line, respectively, were cultured in RPMI 1640 medium supplemented with 10% foetal calf serum and 5 mM L-glutamine (Life Technologies Inc., Paisley, UK). H929, a multiple myeloma cell line, was cultured in the same medium supplemented with 0.02% 2-mercaptoethanol. All cell lines were from ATCC (Middlesex, UK).

### Reagents

ABT-199, A-1331852 and A-1210477 were kindly supplied by Abbvie Inc., (North Chicago, IL, USA). Peptides for BIM (MRPEIWIAQELRR IGDEFNA), BAD (LWAAQRYGRELR RMSDEFEGSFKGL), MS-1 (RPEIWMTQGLRRLGDEINAYYAR), HRK (WSSAAQLTAARLKALGDELHQ) and PUMA-2A (EQWAREIGAQARRMAADLNA) were from New England Peptide (Gardner, MA, USA) or GenScript (Piscataway, NJ, USA). Other reagents were from Sigma-Aldrich Co. (St. Louis, MO, USA).

### BH3 profiling, DBP and apoptosis

For BH3 profiling, cells were permeabilised with digitonin (0.002%) and loss of mitochondrial membrane potential (*ψ*_m_) assessed using TMRE (200 nM) after incubation with BH3 peptides as described (Ryan and Letai, 2013). For DBP, cells were incubated for 1 h with A-1331852 (1 *μ*M), A-1210477 (10 *μ*M) or ABT-199 (1 *μ*M) prior to incubation with BAD or MS-1 peptide (10 *μ*M) for 2 h and assessment of *ψ*_m_. Apoptosis was quantified by measuring phosphatidylserine externalisation (Vogler *et al*, 2009b).

## Results

**Figure 1:**
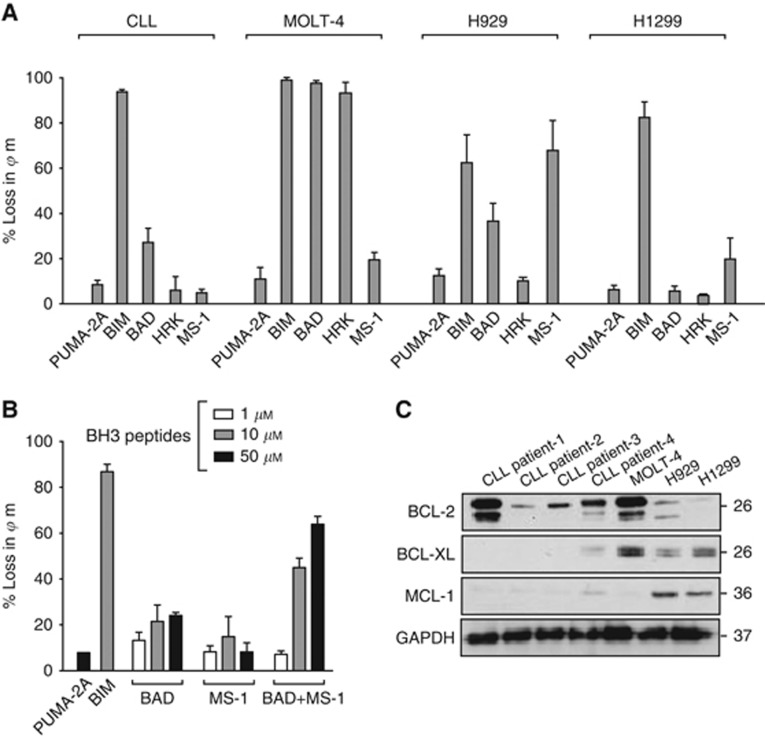
**BH3 profiling in cell lines.** (**A**) Cells were incubated with BH3 peptides (10 *μ*M) in TEB buffer (containing 0.002% digitonin) for CLL cells (30 min), MOLT-4 cells (1 h), and H929 and H1299 cells (2 h). Mitochondrial potential was assessed and changes calculated with reference to DMSO & FCCP-treated cells. Data represents the Mean±s.e.m. of triplicate experiments. (**B**) Mitochondrial depolarisation of CLL cells exposed to BAD and MS-1 peptides alone or in combination. (**C**) Western blots of either CLL cells from four patients or the cell lines were analysed for expression of the indicated proteins. No detectable BCL-w or BFL-1 was observed in any of the cells.

To validate the efficacy of BH3 profiling on cells addicted to specific BCL-2 family members, primary CLL cells, addicted to BCL-2 (Del Gaizo Moore *et al*, 2007; Vogler *et al*, 2009a, 2009b), MOLT-4 and H929 cell lines, addicted to BCL-X_L_ (Leverson *et al*, 2015a) and MCL-1 (Leverson *et al*, 2015b), respectively, and H1299 cells addicted to both BCL-X_L_ and MCL-1 (Varadarajan *et al*, 2013) were selected. For initial BH3 profiling experiments, BIM was used as a positive control due to its ability to interact with all anti-apoptotic members and PUMA-2A was a negative control. HRK and MS-1 peptides react exclusively with BCL-X_L_ and MCL-1, respectively, and BAD peptide reacts with BCL-2, BCL-X_L_ and BCL-w (Ryan and Letai, 2013; Foight *et al*, 2014). BH3 profiling showed that all the cells were primed for mitochondrial-dependent cell death by their sensitivity to BIM peptide. Chronic lymphocytic leukaemia cells were sensitive to BIM and BAD peptides but not to HRK or MS-1, consistent with their BCL-2-addiction. However, as the BAD peptide was much less efficient than the BIM peptide, it suggested that CLL cells may depend on additional BCL-2 family proteins for survival in scenarios, such as an alteration in tissue microenvironment (Vogler *et al*, 2009a). Some support for this was provided by the increased mitochondrial depolarisation observed following combination of BAD and MS-1 peptides (Figure 1B). However, the CLL cells used in this study from four different patients predominantly expressed BCL-2 with little or no BCL-X_L_ or MCL-1 (Figure 1C). In contrast, MOLT-4 cells exhibited sensitivity to BIM, BAD and HRK but not MS-1, suggesting their dependence on BCL-X_L_ and not MCL-1 for survival (Figure 1A). BH3 profiling of H929 cells revealed sensitivity to BIM and MS-1 peptides, in agreement with their reported addiction to MCL-1, although some sensitivity to BAD was also observed (Figure 1A). Only BIM induced significant mitochondrial depolarisation in H1299 cells (Figure 1A), suggesting that H1299 cells depended on more than one BCL-2 family member for survival. What regulates the dependency of these cell types on a specific BCL-2 family member is still unknown, as in addition to expressing the expected anti-apoptotic member required for their survival, namely BCL-2 for CLL, BCL-X_L_ for MOLT-4 and MCL-1 for H929, other anti-apoptotic proteins were also expressed (Figure 1C) in agreement with earlier studies (Del Gaizo Moore *et al*, 2007, 2008).

**Figure 2:**
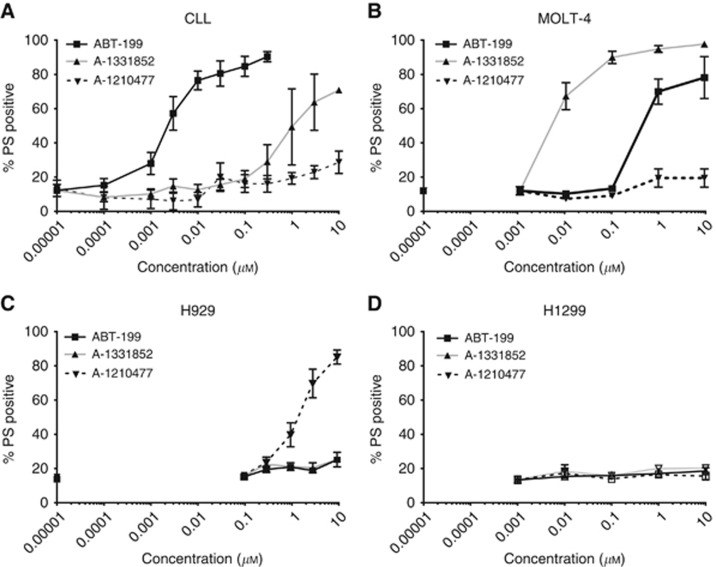
**BH3-mimetic-induced apoptosis in (**A**) CLL, (**B**) MOLT-4, (**C**) H929 and (**D**) H1299 cells.** Cells were incubated with ABT-199 (BCL-2 inhibitor), A-1331852 (BCL-X_L_ inhibitor) and A-1210477 (MCL-1 inhibitor) for 4 h and apoptosis determined. Data represents the mean±s.e.m. of triplicate experiments.

BH3 profiling offers insight into the BCL-2 family dependency of cancer cells and has been particularly valuable, while a limited range of selective potent BCL-2 family inhibitors was available. However, since the recent discovery of specific inhibitors of BCL-X_L_ and MCL-1, we questioned if the inhibitor toolkit comprising ABT-199, A-1331852 and A-1210477 (targeting BCL-2, BCL-X_L_ and MCL-1, respectively) might be sufficient to draw similar conclusions. To accomplish this, apoptosis was assessed in response to these inhibitors. Chronic lymphocytic leukaemia cells were exquisitely sensitive to ABT-199 (IC_50_=2.5 nM after 4-h exposure) but insensitive to A-1210477 and to a great extent to A-1331852 (IC_50_=1.1 *μ*M) (Figure 2), thus confirming that CLL cells are addicted to BCL-2. MOLT-4 cells were sensitive only to A-1331852 (IC_50_ ∼6.7 nM), supporting their dependence on BCL-X_L_, whereas H929 were only sensitive to A-1210477 (IC_50_=1.8 *μ*M), confirming their MCL-1 addiction (Figure 2). In agreement with BH3 profiling, H1299 cells were insensitive to all three inhibitors alone (Figure 2), suggesting a possible dependence on more than one anti-apoptotic BCL-2 family member.

**Figure 3:**
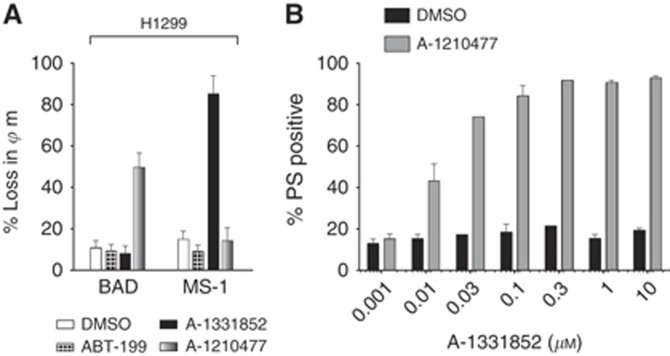
**Synergy in H1299 cells.** H1299 cells (**A**) were incubated for 1 h with ABT-199 (1 *μ*M), A-1331852 (1 *μ*M) or A-1210477 (10 *μ*M) prior to profiling with either BAD or MS-1 (10 *μ*M) peptide for 2 h and loss of *ψ*_m_ measured. (**B**) H1299 cells were coincubated with A-1210477 (10 *μ*M) and with A-1331852 for 4 h before apoptosis was measured. Data represents the mean±s.e.m. of triplicate experiments.

To assess if multiple BCL-2 family members regulated apoptosis in H1299 cells, we carried out DBP following exposure of cells to the specific inhibitors for 1 h prior to BH3 profiling with BAD or MS-1 peptide. In H1299 cells, exposure to A-1210477 but not ABT-199 or A-1331852 increased sensitivity to BAD, confirming a dependency on MCL-1 and either BCL-2 and/or BCL-X_L_ for survival (Figure 3A). To distinguish between BCL-2 and BCL-X_L_, we carried out similar treatments with the inhibitors but profiled using MS-1. Prior exposure to A-1331852 but not ABT-199 or A-1210477 sensitised cells to MS-1 (Figure 3A). Taken together, these results suggested that H1299 cells depended on both MCL-1 and BCL-X_L_ for survival, which was further confirmed by the extensive apoptosis observed following a combination of A-1331852 and A-1210477 (Figure 3B). Following inhibition of MCL-1 in H1299 cells, the apparent IC_50_ for A-1331852 was 1.4 nM, which was similar to that observed in MOLT-4 cells.

## Discussion

Assessment of mitochondrial perturbation in response to different BH3 peptides, clearly provides valuable mechanistic insights into mitochondrial alterations that affect apoptotic signalling, whereas the cell-permeable chemical inhibitor toolkit gives a simple direct readout of apoptosis from a simple cell culture system. Limitations of BH3 profiling include some difficulty in choosing optimal peptide concentrations, whereas current limitations of the inhibitor toolkit include the lack of specific inhibitors for the less common anti-apoptotic BCL-2 family members, such as BCL-2-A1 and BCL-w, as well as the lower potency of the MCL-1 inhibitor compared with those for BCL-2 and BCL-X_L_. Currently there are major efforts to develop more specific and potent inhibitors of MCL-1, as MCL-1 is a major resistance factor to ABT-263-induced apoptosis (van Delft *et al*, 2006; Zhang *et al*, 2011; Gores and Kaufmann, 2012). In some cases we observed conflicting results between BH3 profiling and the inhibitor toolkit, for example, the BAD peptide induced mitochondrial depolarisation in H929 cells implying a possible BCL-2/BCL-X_L_ dependence (Figure 1A), whereas the inhibitor data indicated MCL-1 dependence of the cells (Figure 2), in agreement with other data in the literature (Leverson *et al*, 2015b). Although the reason for this is unclear, it may indicate a limitation of using 23mer peptides, which can only partially mimic the endogenous protein. This together with some promiscuity of the peptides indicates that the inhibitor toolkit may ultimately be a more reliable predictor of tumour sensitivity than BH3 profiling, although this requires further validation. From a therapeutic perspective, it is most attractive to treat tumours with a specific BCL-2 family inhibitor, as that will lessen the likelihood of non-specific toxicities, although this may result in a more rapid development of resistance. Thus both BH3 profiling and the selective chemical inhibitor toolkit successfully identified cells dependent on a single anti-apoptotic BCL-2 family member for survival (Figures 1 and 2). However, most cancer cells probably depend on more than one anti-apoptotic protein for survival. In this regard, the DBP experiments are instructive. A short exposure (1 h) to A-1331852 and A-1210477 sensitised H1299 cells to MS-1 and BAD peptide, respectively (Figure 3A). Besides clearly demonstrating the dependency of H1299 cells on both BCL-X_L_ and MCL-1 for survival, these results illustrate how rapidly the inhibitors penetrate cells and inhibit their target proteins. Our results highlight the ability of the inhibitor toolkit to provide a rapid, inexpensive and simple means to assess the chemosensitivity of tumour cells and in conjunction with DBP to facilitate the optimisation of individualised therapy.
